# Antenatal diagnosis of fetal umbilical hernia on mid-trimester sonography

**DOI:** 10.1093/bjrcr/uaag020

**Published:** 2026-05-28

**Authors:** Sreedevi Sathian

**Affiliations:** Department of Radiology, Koya’s Hospital, Kozhikode 673631, Kerala, India

**Keywords:** congenital hernia of the umbilical cord, fetal umbilical hernia, antenatal ultrasound, abdominal wall defect, mid-trimester anomaly scan

## Abstract

Congenital hernia of the umbilical cord (CHUC) is a rare and often under-recognized anterior abdominal wall defect that is frequently misdiagnosed as an omphalocele. It represents a distinct embryological entity with a benign course and favorable prognosis. Prenatal ultrasonography is crucial for accurate diagnosis, appropriate parental counseling, and safe perinatal management, particularly to prevent bowel injury during umbilical cord clamping. We report a case of isolated fetal umbilical hernia detected on a routine mid-trimester anomaly scan and highlight key sonographic features that aid differentiation from other abdominal wall defects.

## Clinical presentation

A 20-week gestation primigravida underwent a routine mid-trimester fetal anomaly scan. Ultrasound examination demonstrated a small probable midline anterior abdominal wall defect at the level of the umbilical ring.

## Imaging findings

**Figure 1:**
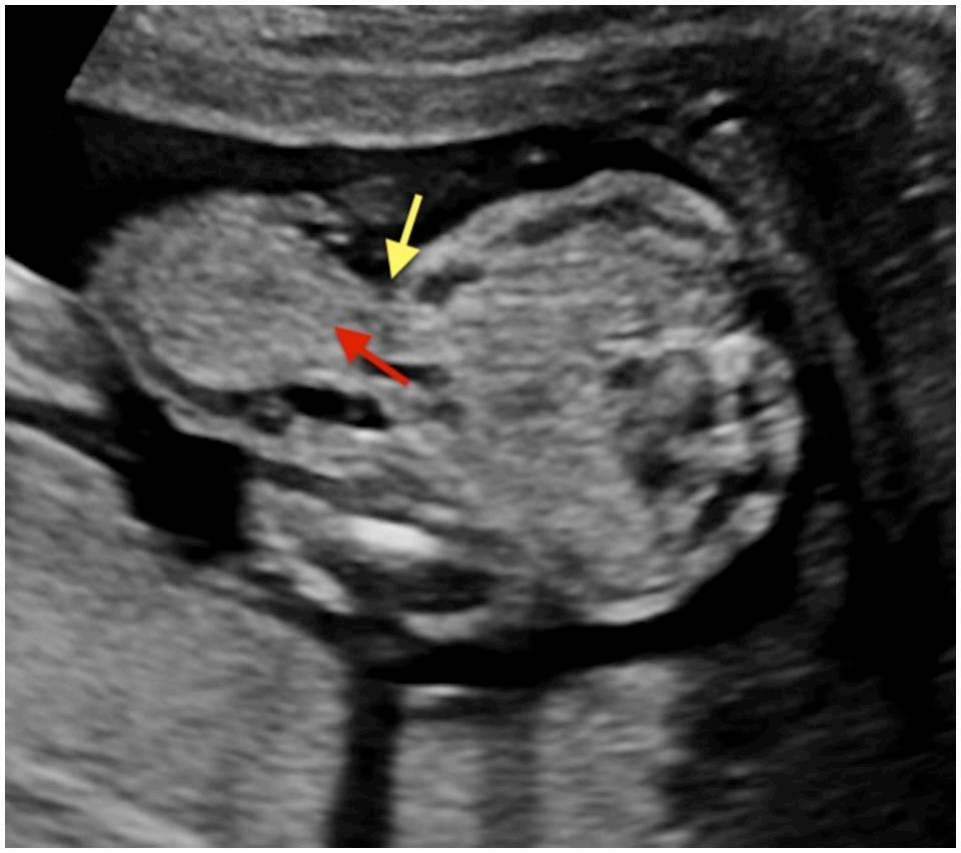
A mid-trimester obstetric ultrasound, shows a small midline anterior abdominal wall defect (red arrow) at the umbilical ring with herniation of bowel loops into the umbilical cord. The herniated contents are covered by intact skin, and no liver tissue is present—features consistent with a congenital hernia of the umbilical cord. A normally inserted umbilical cord (yellow arrow) distinct from the bowel-containing sac, helping differentiate this condition from omphalocele, where the cord inserts at the apex of the sac is visible.

**Figure 2:**
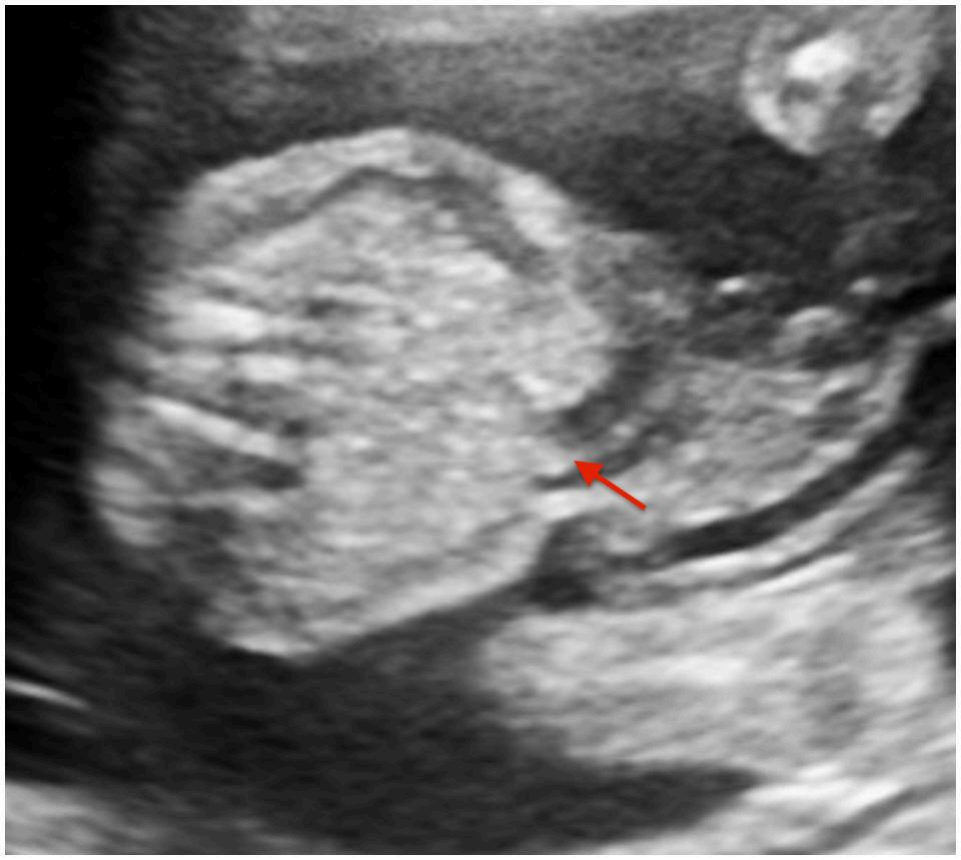
Ultrasound image of the fetal anterior abdominal wall demonstrating skin-covered herniation of bowel loops through a narrow umbilical defect with normal cord insertion (red arrow). The absence of bowel dilatation or abnormal echogenicity suggests preserved bowel viability.

**Figure 3:**
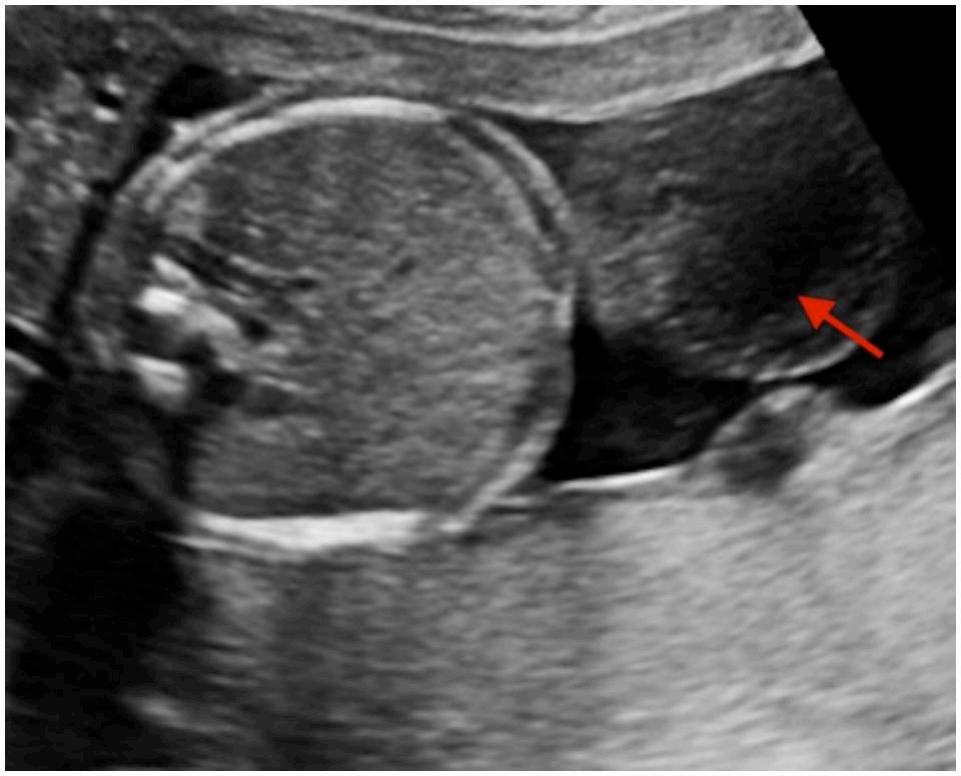
Antenatal ultrasound image demonstrating a Wharton’s jelly cyst (red arrow) within the umbilical cord in the same case of congenital hernia of the umbilical cord, seen as a well-defined anechoic cystic lesion without internal vascularity, distinct from the umbilical vessels.

A sac-like protrusion containing bowel loops was identified, with complete skin coverage over the herniated contents. The umbilical cord insertion was normal and clearly separate from the hernia sac. The herniated bowel loops showed normal echogenicity, with no evidence of dilatation, vascular compromise, or ischemic changes. No liver tissue was identified within the sac. Fetal biometric parameters, including abdominal circumference, were appropriate for gestational age. A detailed anatomical survey did not reveal any associated structural anomalies, and the amniotic fluid volume was within normal limits (Figures 1-2). There was also an incidental finding of a Wharton’s jelly cyst, which contains a degenerated gelatinous substance protecting the umbilical vessels (Figure 3). Based on the imaging findings, a diagnosis of isolated congenital hernia of the umbilical cord (CHUC) was made.

## Outcome

The parents were counselled regarding the benign nature of the condition and its favorable postnatal outcome. A healthy male infant with a CHUC was delivered. A pediatric surgery consultation was obtained, and reduction surgery was planned for a later date.

## Discussion

Fetal anterior abdominal wall defects comprise a spectrum of congenital anomalies with varying prognostic implications. Congenital hernia of the umbilical cord is a rare and benign condition that is frequently overlooked or misdiagnosed on prenatal imaging due to its resemblance to omphalocele and other ventral abdominal wall defects.^1^^,^^2^ Accurate prenatal identification is crucial, as CHUC carries an excellent prognosis and typically requires minimal postnatal intervention.^3^

Unlike omphalocele, CHUC is not associated with chromosomal abnormalities and is characterized by herniation of abdominal contents into a normally inserted umbilical cord, with intact skin covering the defect.^4^ Misclassification may result in unnecessary parental anxiety, altered pregnancy management, and preventable perinatal complications. This case report emphasizes the importance of recognizing CHUC during routine antenatal ultrasounds and reviews its distinguishing imaging features.

Congenital hernia of the umbilical cord results from incomplete return of physiologically herniated midgut loops into the abdominal cavity during early embryogenesis. Normally, midgut herniation occurs between 5 and 10 weeks of gestation, with return and fixation completed by 11-12 weeks. Failure of this process is believed to result in CHUC.^5^

CHUC can be reliably differentiated from omphalocele by careful evaluation of the umbilical cord insertion and the covering of the herniated contents. In CHUC, the umbilical cord inserts normally at the abdominal wall, and the herniated bowel is covered by skin extending up to the neck of the sac. In contrast, omphalocele presents as a larger defect involving skin and muscle, with the umbilical cord inserting at the apex of a membranous sac and a high association with chromosomal abnormalities, reported in up to 30%-40% of cases.^4^^,^^6^

Mirza et al. proposed a classification system for CHUC based on morphology and clinical presentation: type 1 (simple hernia without complications), type 2 (associated with intestinal obstruction), type 3 (associated with mucosal prolapse), and type 4 (associated with bowel evisceration).^7^ Although gastrointestinal involvement is most common, occasional associations with extraintestinal anomalies have been described.^8^

Prenatal ultrasonography remains the cornerstone of diagnosis. High-resolution transvaginal ultrasound may detect CHUC as early as 12-14 weeks of gestation, although detection is operator dependent.^9^ Accurate antenatal diagnosis is particularly important to prevent iatrogenic bowel injury during umbilical cord clamping at delivery, a complication that has been reported in undiagnosed cases.^2^

Fetal MRI may serve as a complementary modality in selected cases when sonographic findings are inconclusive or when complex ventral abdominal wall anomalies are suspected, owing to its superior soft-tissue contrast and multiplanar capability.^10^

## Learning points

- Congenital hernia of the umbilical cord is a benign fetal anomaly with minimal chromosomal association and an excellent prognosis.
- Accurate antenatal identification through meticulous evaluation of the umbilical cord insertion and defect morphology is essential to differentiate CHUC from other anterior abdominal wall defects like omphalocele.
- Increased awareness of this condition facilitates appropriate prenatal counseling, safe delivery planning, and optimal postnatal management.
